# Prescriptions

**Published:** 1895-05

**Authors:** 


PRURITUS VULVÆ.

Dr. B. F. Baer has found the following combination to be the best palliative for pruritus occurring at the menopause:

℞ Morphine sulphate.....6 grains.

Boric acid,...............1½ ʒ.

Camphor-water......6 fl. ℥.—M.

Label: Poison. Apply to the affected parts after ablution with warm water and Castile soap.—*Philadelphia Polyclinic.*

HEPATIC AND NEPHRITIC COLIC.

℞ Valerianate of amyl,
Sulphuric ether. ......aa gtt. iij.

M. For 1 capsule. Let 20 such capsules be made.

Sig.: Two capsules every half-hour until 6 have been taken.—*La Medecine Moderne.*

STOMATITIS.

In the herpetic form Dr. Marfan speaks favorably of a mixture thus composed:

℞ Distilled water,
Glycerin...............aa f ʒ iiss.
Iodine,
Iodide of potassium....aa gr. vj.

M. Sig.: For local use. Apply to lesions.—*La Medecine Moderne.*

LUMBAGO.

A prescription of Dr. S. Solis-Cohen's is

℞ Sodium salicylate...........½ ℥.

Potassium iodide...........2 ʒ.

Compound syrup of sarsaparilla.........1½ fld. ℥.

Water, sufficient to make...............3 fld. ℥.

M. Sig.: A teaspoonful in water thrice daily, after meals.—*Philadelphia Polyclinic.*

Please mention Southern Medical Record

### FOR AMENORRHŒA.

The following promises well:

℞ Hydrarg. chloridi corrosivi,.................. gr. ¾.
Sodii arseniatis, ............gr. j.
Ferri sulphatis exsiccatæ..................gr. xxx.
Potassii carbonatis........gr. xv.
Extracti nucis vomicæ....................gr. v.
M. Divid. in pil. xxx.

Sig.: One pill to be taken before each meal.—*Medical and Surgical Reporter.*

### PURULENT OPHTHALMIA.

Cesaris has found the following formula useful in purulent ophthalmia, affections of the cornea, syphilides, etc.:

℞ Salicylate of cadmium...gr. iss.

Distilled water..........f ʒ iiss.

M. Sig.: Use as a collyrium.

For injections in gonorrhœa and vaginitis he employs

℞ Salicylate of cadmium...... ʒ ss.

Distilled water. ..... .f ℥ vj.—M.

—*La Medecine Moderne.*

### NOCTURNAL INCONTINENCE OF URINE.

When associated with a rheumatic diathesis, Dr. W. L. Kendall, of Meridian, Miss., writes to the *Louisville Medical Monthly* that a good prescription is

℞ Pot. iod.................. ʒ iiss.

Fld. ext. xanthoxyli,

Fld. ext. stillingiæ,

Fld. ext. fraxini Americanæ,

Fld. ext. sarsaparillæ....aa ℥ ij.

M. Sig.: Two tablespoonfuls in a little water three times a day, and one dose at bedtime. Commence and give one hour before each meal.

### TO PREVENT BED-SORES.

After washing the parts with an antiseptic solution twice daily, dry and apply the following powder:

℞ Pulv. camph. ............ ʒ v.

Amyl. pulv.............. ℥ iiiss.

Cretæ gallic................ ℥ ij.

Alum ustum..... .. ........ ʒ j.

Acid. boracic........ .. .... ℥ j.

Pulv. oxidi zinc............. ℥ j.

Acid. carbolic.............. ʒ ss.

Ol. gaultheria .. .... ʒ ss.—M.

—*Prescription.*

